# The Transfusion of Blood

**Published:** 1852-03

**Authors:** 


### ARTICLE III.

### *The Transfusion of Blood.*

We find in the *Ami de L'Ordre* of Grenoble, of the 30th November, the following cure, as to the authenticity of which we reserve our opinion until further informed :

"For some days the journals of Paris have been very much occupied with a surgical operation, known by the name of the transfusion of blood, which was performed at the Hospital of St. Louis by one of the ablest physicians. The same operation has just succeeded completely in a village near Grenoble. We think it our duty to give certain details of the circumstances attending this operation, which were transmitted to us by a correspondent at Domene.

After an unfortunate accouchment, the wife of Mallet, a butcher at Saucey, aged 30 years, experienced a hæmorrhage so abundant, that in a few minutes she was reduced to extreme feebleness. It was then decided to call in medical aid. Dr. Marmontier of Domine was sent for ; but he did not reach the bedside of the patient until two hours after the accident, when the illness had made considarable progress. The midwife and several other women who surrounded the patient, saw her motionless, without consciousness, and did not doubt for a moment that her death was approaching. The Doctor determined to try the transfusion of blood. He satisfied himself that there was still feeble circulation. Immediately he laid bare the basilic vein of the right arm to the extent of one or two centimeters: he opened it, and inserted the pipe of a small syringe, with all the precaution which the gravity of the circumstances required. A neighbor, Miss Fagnet, consented to be bled. In a few moments, the blood which had been taken from her veins was flowing in those of the patient, and carried new life into her heart, which had almost been stilled. The transfusion was so successful, that in a few moments after Mrs. Mallet was restored to consciousness, and was able to make some slight movements. The cure commenced immediately, and with every prospect of being complete. Her strength returned with astonishing rapidity, and now the woman has entirely recovered her health. Her feebleness was so great at the time of the operation, that she was not aware of it except a kind of tickling in the arm that was incised.—*Jour. des Connaissances.* [*In N. O. Med. Jour.*

